# Evaluating the suitability of hyper- and multispectral imaging to detect foliar symptoms of the grapevine trunk disease Esca in vineyards

**DOI:** 10.1186/s13007-020-00685-3

**Published:** 2020-10-21

**Authors:** Nele Bendel, Anna Kicherer, Andreas Backhaus, Hans-Christian Klück, Udo Seiffert, Michael Fischer, Ralf T. Voegele, Reinhard Töpfer

**Affiliations:** 1 Institute for Grapevine Breeding, Julius Kühn-Institut, Federal Research Centre for Cultivated Plants, Geilweilerhof, 76833 Siebeldingen, Germany; 2 Biosystems Engineering, Fraunhofer Institute for Factory Operation and Automation (IFF), Sandtorstr. 22, 39106 Magdeburg, Germany; 3 Institute for Plant Protection in Fruit Crops and Viticulture, Julius Kühn-Institut, Federal Research Centre for Cultivated Plants, Geilweilerhof, 76833 Siebeldingen, Germany; 4 Institute of Phytomedicine, University of Hohenheim, Otto-Sander-Straße 5, 70599 Stuttgart, Germany

**Keywords:** Plant phenotyping, Grapevine trunk disease, Disease detection, Spectral imaging, Phenoliner, Phenotyping platform, Precision viticulture

## Abstract

**Background:**

Grapevine trunk diseases (GTDs) such as Esca are among the most devastating threats to viticulture. Due to the lack of efficient preventive and curative treatments, Esca causes severe economic losses worldwide. Since symptoms do not develop consecutively, the true incidence of the disease in a vineyard is difficult to assess. Therefore, an annual monitoring is required. In this context, automatic detection of symptoms could be a great relief for winegrowers. Spectral sensors have proven to be successful in disease detection, allowing a non-destructive, objective, and fast data acquisition. The aim of this study is to evaluate the feasibility of the in-field detection of foliar Esca symptoms over three consecutive years using ground-based hyperspectral and airborne multispectral imaging.

**Results:**

Hyperspectral disease detection models have been successfully developed using either original field data or manually annotated data. In a next step, these models were applied on plant scale. While the model using annotated data performed better during development, the model using original data showed higher classification accuracies when applied in practical work. Moreover, the transferability of disease detection models to unknown data was tested. Although the visible and near-infrared (VNIR) range showed promising results, the transfer of such models is challenging. Initial results indicate that external symptoms could be detected pre-symptomatically, but this needs further evaluation. Furthermore, an application specific multispectral approach was simulated by identifying the most important wavelengths for the differentiation tasks, which was then compared to real multispectral data. Even though the ground-based multispectral disease detection was successful, airborne detection remains difficult.

**Conclusions:**

In this study, ground-based hyperspectral and airborne multispectral approaches for the detection of foliar Esca symptoms are presented. Both sensor systems seem to be suitable for the in-field detection of the disease, even though airborne data acquisition has to be further optimized. Our disease detection approaches could facilitate monitoring plant phenotypes in a vineyard.

## Background

The Esca complex—along with Botryosphaeria dieback and Eutypa dieback—is one of the most important GTDs worldwide causing significant economic losses annually [1]. A complex group of wood deteriorating fungi is assumed to be involved in disease development. The pathogens infect vines especially through pruning wounds in the field, but can sometimes also be found in nurseries. Thus, the dissemination with young grafted vines cannot be excluded [2]. The only effective treatment known against these fungi was sodium arsenite, but after its ban, because of its toxicity to humans and the environment, no curative treatment is available. Even though many research efforts were made to find new solutions for plant protection, at the moment, only prophylactic measures, such as *Trichoderma* species, can be applied to reduce the spread of the disease [2, 3].

Although grapevine cultivars differ in their sensitivity to the disease complex, no cultivated or wild grapevine species is known to be resistant [4, 5]. Typical wood symptoms are several forms of discolorations and necroses, vascular infections, and in older vines also white rot [6]. Since the Esca complex is a slow perennial disease, external symptoms do usually not become apparent until several years after infection [7]. External symptoms can occur either slow, developing in a so-called chronic form, or in an acute apoplectic form, eventually leading to the death of infested vines. Foliar symptoms of the chronic form are characterized by interveinal necrosis and yellow or red chlorosis producing a tiger-stripe pattern, which can appear on single leaves or shoots as well as on multiple shoots [8]. So far, no correlation between the severity of wood symptoms and the expression of leaf symptoms could be shown [9]. Since chronic symptoms do not develop consecutively, their appearance cannot be predicted from year to year [7]. Therefore, an annual monitoring becomes fundamental to estimate the real incidence of the disease in a vineyard.

Traditional monitoring depends on visual ratings by experts, thus, having the disadvantage of being time consuming and subjective. In the last years, several sensor-based phenotyping methods have been established to overcome the phenotyping bottleneck [10], but also for the development of new management strategies in precision agriculture [11]. The application of sensor technology ensures the non-invasive and objective assessment of various plant traits on different scales ranging from leaf level via plants up to whole fields [12–15]. Since analysis can be automated, a higher throughput is possible in comparison to traditional phenotyping approaches. One of the most promising techniques for disease detection is the employment of spectral sensors, which are able to detect biochemical and biophysical changes in plants upon an infection by capturing changes in their reflectance [11]. Unlike RGB cameras they do not only measure reflectance in the visible range of light (VIS; 400–700 nm), but also in the near infrared (NIR; 700–1000 nm) and even the short wave infrared (SWIR; 1000–2500 nm), thereby providing a much higher informational content. Reflectance can either be recorded in the whole spectral region (hyperspectral) or at selected spectral bands only (multispectral) [16]. Several studies have shown that these sensor techniques have a high potential for the detection of plant diseases [17–21].

In recent years, the sensor-based detection of various grapevine diseases has become a progressive field of research. Symptomatic grapes and leaves infected with *Erysiphe necator* [22, 23] and *Plasmopara viticola* [24], the causal agents of powdery and downy mildew, respectively, have been analyzed under laboratory conditions. Furthermore, intensive studies concerning the detection of grapevine leafroll disease (GLD) [25–27] as well as of grapevine yellows (GY) [28, 29] have been performed and also foliar Esca symptoms were examined in different proximal and remote sensing approaches. For the detection of chronic Esca symptoms both Al-Saddik et al. [30] and Junges et al. [31] used a portable spectroradiometer (350–2500 nm) to collect hyperspectral reflectance data of symptomatic and asymptomatic leaves under laboratory conditions. While Junges et al. [31] measured and calculated the chlorophyll index per leaf to discriminate different symptom intensities; Al-Saddik et al. [30] combined the spectral data with textural data gained from RGB images. Further analyses were performed by Gallo et al. [32] and Rançon et al. [33] both using platform prototypes for the in-field detection of Esca leaf symptoms. Two platform prototypes developed by Gallo et al. [32] were each equipped with multispectral cameras calculating the Normalized Difference Vegetation Index (NDVI) for disease detection and one vehicle was additionally equipped with LiDAR sensors to gain information on canopy thickness. Rançon et al. [33] compared different algorithms and methods for a classification on both leaf and plant scale on the basis of digital images. In an airborne approach, Kerckech et al. [34] also worked with RGB images combining deep learning with different color spaces and vegetation indices for vine disease detection in manually annotated data. Similar studies were conducted by Di Gennaro et al. [35] and Albetis et al. [36] both using unmanned aerial vehicles (UAVs) to obtain multispectral images calculating the NDVI and various other vegetation indices. Di Gennaro et al. [35] were even able to detect infected vines two weeks before symptom development.

This study focuses on the suitability of hyper- and multispectral imaging for the in-field detection of foliar Esca symptoms over three consecutive years. For this purpose, ground based hyperspectral data in the range of 400–2500 nm were analyzed to (i) develop a disease detection model for the discrimination between symptomatic and asymptomatic grapevines using both original field data as well as manually annotated data, (ii) test the potential early detection of the disease, and (iii) identify relevant wavelengths for the simulation of a multispectral approach. Finally, this multispectral simulation is compared to real multispectral data collected by a UAV in order to evaluate the applicability of the two different systems.

## Results

### Comparison of original and annotated data

Disease detection models were developed individually for original field data as well as annotated data to evaluate the influence of heterogeneous field data on model performance. Field data is usually a mixture of symptomatic and asymptomatic leaves as Esca symptoms do not develop uniformly on the plant, which may influence a disease detection model. In order to simulate the best possible conditions, symptomatic leaves were labeled manually and then used for the development of a second disease detection model.

Table 1 shows the Classification Accuracy (CA), True Positive Rate (TPR) and False Positive Rate (FPR) for the detection of Esca leaf symptoms for both models and individual years. Regarding original data, TPRs of 70 to 82% per year were achieved depending on the camera, meaning that an acceptable amount of pixels was correctly identified. However, 20 to 34% of the pixels were falsely classified as positives. This results in CAs of 73, 70, and 77% (VNIR) and 73, 81, and 80% (SWIR) for the years 2016, 2017, and 2018, respectively. When only symptomatic leaves were considered, results improved significantly. TPRs of up to 100% could be reached with satisfying FPRs of 0 to 11%, leading to CAs between 88 and 95%. Since no differences could be detected between the VNIR and SWIR range neither for original nor annotated data, both wavelength ranges seem to be suitable for the differentiation tasks**.**

**Table 1:** Results for the detection of Esca leaf symptoms using original field data and annotated data

				VNIR			SWIR	
			2016	2017	2018	2016	2017	2018
Modeling	Original data	CA (%)	73 ± 2	70 ± 2	77 ± 2	73 ± 2	81 ± 2	80 ± 2
		TPR (%)	71 ± 2	73 ± 2	72 ± 2	70 ± 10	82 ± 5	74 ± 10
		FPR (%)	29 ± 2	32 ± 2	22 ± 2	34 ± 9	26 ± 4	20 ± 7
	Annotated data	CA (%)	92 ± 1	90 ± 1	94 ± 1	88 ± 1	95 ± 1	92 ± 1
		TPR (%)	89 ± 1	90 ± 1	93 ± 1	86 ± 4	90 ± 1	100 ± 1
		FPR (%)	0 ± 1	11 ± 1	5 ± 1	2 ± 7	6 ± 1	5 ± 1
Application per plant	Original data	CA (%)	81	73	88	74	84	95
		TPR (%)	79	76	86	63	80	86
		FPR (%)	19	27	12	23	16	5
	Annotated data	CA (%)	78	75	91	79	91	90
		TPR (%)	58	71	71	60	60	71
		FPR (%)	17	25	72	21	8	8

For modeling, all pixels were evaluated not considering spatial scales. Developed models were then applied on plant scale using all leaves for majority voting

*CA * classification accuracy, *TPR * true-positive rate, *FPR * false-positive rate

The model development is based on an evaluation of all pixels not considering spatial scales. In a next step, the detection models were therefore applied on plant scale. For these evaluations, a majority voting of pixel results can be used to derive a prediction of the symptom status per plant. The number of false positives can be manually adjusted, thereby, influencing the number of true positives. For the per plant evaluation, FPR was set between 5 and 27% in order to achieve the best TPR. Both models showed good CAs of up to 95%, when applied on plant level, but the model developed with original field data performed better, which is indicated by higher TPRs (see Table 1).

### Testing the transferability of disease detection models

In order to test the suitability of disease detection models for practical application, models were developed using two-year hyperspectral data. These models were then applied to the third year, which was not included in the model development. Thereby, the transferability of such disease detection models to unknown data could be examined.

Performances of the different models using original field data as well as annotated data are shown in Table 2. The development of disease detection models for two years showed moderate CAs that were comparable to the models developed on one experimental year. Again, the model using annotated data performed better. For the original field data, TPRs of 71, 73, and 72% (VNIR) and 79, 67, and 79% (SWIR) were achieved for the three models, respectively, with FPRs of 29, 32, and 22% (VNIR) and 36, 25, and 25% (SWIR). This results in CAs of 68, 72, and 72% for VNIR and 72, 73, and 79% for SWIR. Regarding annotated data, higher performances could be obtained; 86 to 90% of all pixels were correctly identified as symptomatic in the VNIR range and 82 to 90% in the SWIR range. Only 6 to 18% of all pixels were falsely classified as symptomatic, leading to CAs of 86 to 93% depending on the model and camera.

**Table 2:** Results for the transferability evaluation of disease detection models

				VNIR			SWIR	
			2016/17	2016/18	2017/18	2016/17	2016/18	2017/18
Modeling	Original data	CA (%)	68 ± 1	72 ± 1	72 ± 1	72 ± 1	73 ± 1	79 ± 1
		TPR (%)	71 ± 4	73 ± 3	72 ± 4	79 ± 8	67 ± 5	79 ± 4
		FPR (%)	29 ± 4	32 ± 3	22 ± 4	36 ± 7	25 ± 4	25 ± 5
	Annotated data	CA (%)	86 ± 2	89 ± 1	89 ± 0	86 ± 1	86 ± 3	93 ± 0
		TPR (%)	86 ± 3	89 ± 3	90 ± 1	83 ± 3	82 ± 4	90 ± 2
		FPR (%)	18 ± 4	12 ± 1	10 ± 1	14 ± 3	13 ± 2	6 ± 2
Application on third year	Original data	CA (%)	63	62	57	57	62	52
		TPR (%)	61	61	47	38	71	51
		FPR (%)	19	43	68	35	30	72
	Annotated data	CA (%)	82	73	59	36	56	49
		TPR (%)	85	79	92	47	42	99
		FPR (%)	14	16	40	20	31	72

For modeling, all pixels were evaluated not considering spatial scales. Developed models were then applied on plant scale using all leaves for majority voting

*CA*  classification accuracy, *TPR*  true-positive rate, *FPR* false-positive rate

The application of the 2 year models to the third year led to very divergent results. Although similar CAs could be reached for the models using original data, the TPRs and FPRs differed significantly, especially in the SWIR range. Here, TPRs between 38 and 71% and FPRs of 30 to 72% were obtained. Models using annotated data performed significantly better for VNIR with TPRs of 85, 79, and 92% and corresponding FPRs of 14, 16, and 40%. Again, the three models showed high variations in the SWIR range. In general, the model 2017/18 performed worst when applied to the third year for both original and annotated data approaches, as indicated by high FPRs. Results also indicate that VNIR appears to provide the more stable wavelength set.

### Pre-symptomatic detection

For the pre-symptomatic detection, in 2017 and 2018, plants were considered that did not show any symptoms at the time of hyperspectral imaging but expressed Esca symptoms within the next 2 weeks. As symptoms were invisible during data acquisition, it was impossible to annotate them manually. Furthermore, these pre-symptomatic leaves could not be matched with symptomatic leaves during the following imaging time points due to vineyard management practices. Therefore, models were developed using original field data only. But again, these models were developed per year for all pixels combined and then applied on plant level.

Table 3 shows CAs as well as TPRs and FPRs for 2017 and 2018. In 2017, CAs of 62 and 74% for VNIR and SWIR, respectively, and similar TPRs could be achieved. However, one third of the pixels were falsely identified as symptomatic. The CAs as well as the TPRs were higher in 2018. Concurrently, the FPRs were lower at 28% (VNIR) and 21% (SWIR), meaning that the pre-symptomatic disease detection performed better in 2018. In general, applying the models per plant higher CAs could be reached. In 2018, better results were achieved again with TPRs of 100% for both cameras and only 20 and 9% false positives for VNIR and SWIR, respectively. For both approaches, the SWIR range seemed to be more suitable. So far, results indicate that pre-symptomatic disease detection should be possible.

**Table 3:** Results for the pre-symptomatic detection of Esca leaf symptoms

		VNIR		SWIR	
		2017	2018	2017	2018
Modeling	CA (%)	62 ± 2	79 ± 2	74 ± 1	86 ± 2
	TPR (%)	63 ± 2	83 ± 4	78 ± 5	87 ± 4
	FPR (%)	35 ± 1	28 ± 3	32 ± 5	21 ± 4
Application per plant	CA (%)	73	81	79	91
	TPR (%)	69	100	75	100
	FPR (%)	26	20	21	9

For modeling, all pixels were evaluated not considering spatial scales. Developed models were then applied on plant scale using all leaves for majority voting

*CA * classification accuracy, *TPR*  true-positive rate, *FPR* false-positive rate.

### Multispectral simulation

**Fig. 1:**
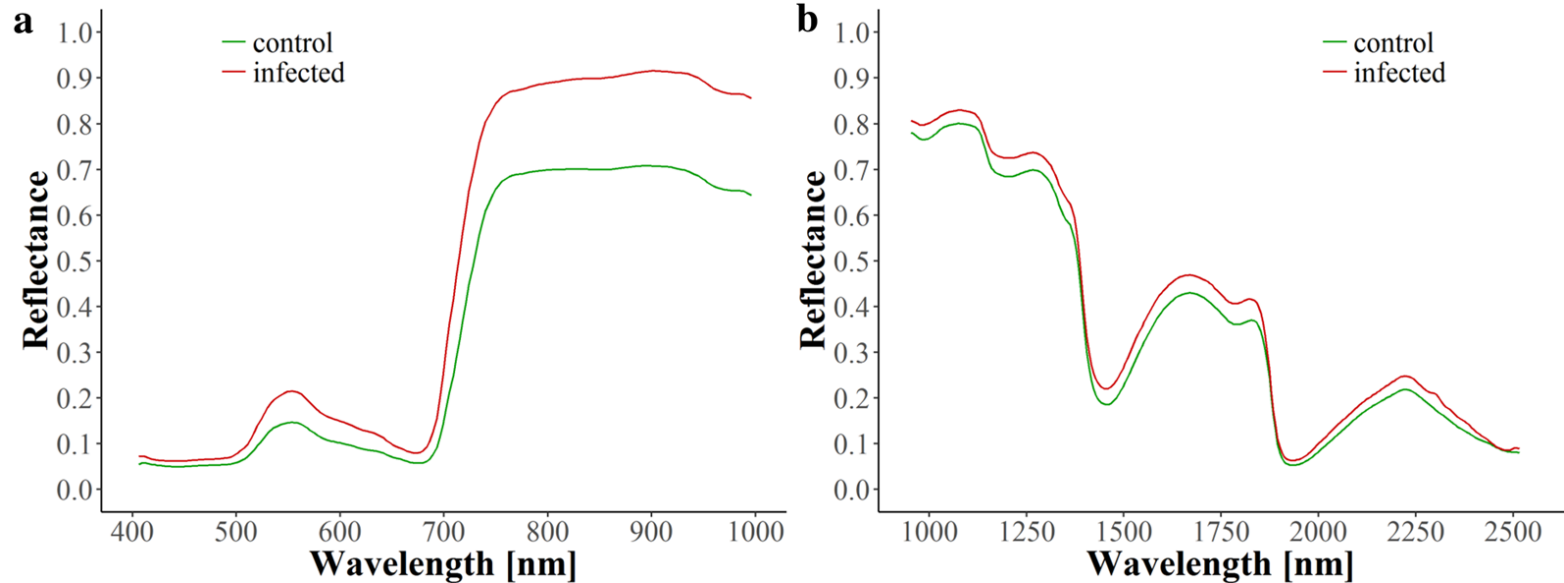
Spectral reflectance corresponding to the average of control (green) and infected (red) annotated leaves in 2017 for VNIR (**a**) and SWIR (**b**)

Exemplarily mean spectra of healthy (control) and symptomatic (annotated) leaves in 2017 are depicted in Fig. 1 (further mean spectra can be found in Additional File 1). When comparing spectral signatures of healthy and infected leaves, clear differences can be seen. Thus, spectra were obviously affected by the Esca disease, which allowed for the differentiation of healthy and infected plants that was presented in the previous paragraphs.

**Fig. 2:**
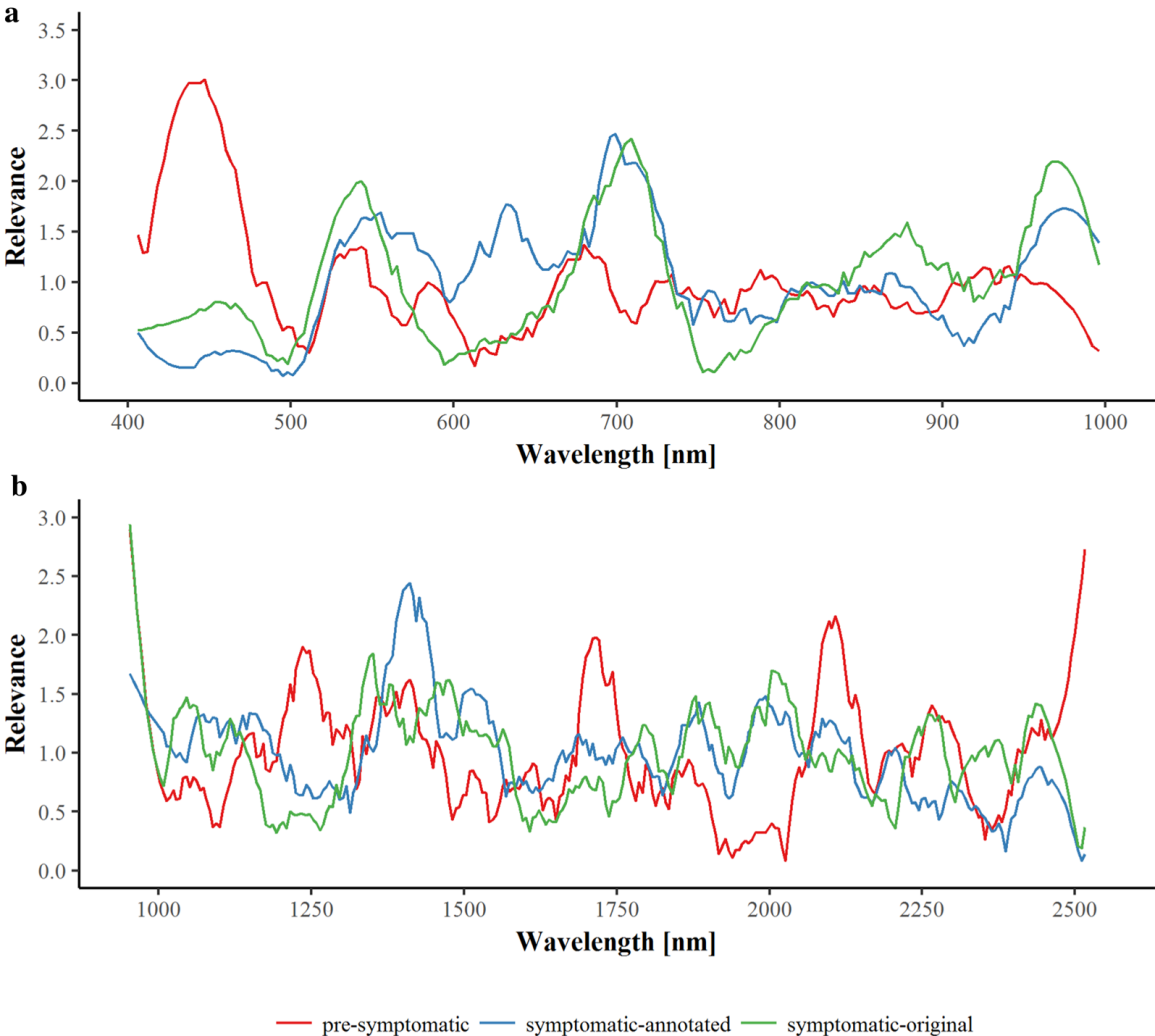
Relevance profiles for the differentiation tasks in the VNIR (**a**) and SWIR (**b)** range combined for the years 2016, 2017, and 2018. Initially, all relevance values are set to 1.0, a number higher than 1.0 indicates an above neutral importance and a number smaller than 1.0 indicates an importance lower than the start condition. The sum of relevance values is constrained to a fixed value so a winner takes all competition for feature importance is built into the system

In order to determine the most important wavelengths for the differentiation task, the machine learning approach enables the calculation of relevance profiles. The relevance profiles for the symptomatic—original and annotated data—and the pre-symptomatic detection models for the combined years are depicted in Fig. 2. Depending on the differentiation task, individual spectral bands are of higher importance. Relevance profiles of the symptomatic detection show almost similar curves for original and annotated data, in contrast to the pre-symptomatic differentiation task.

**Fig. 3:**
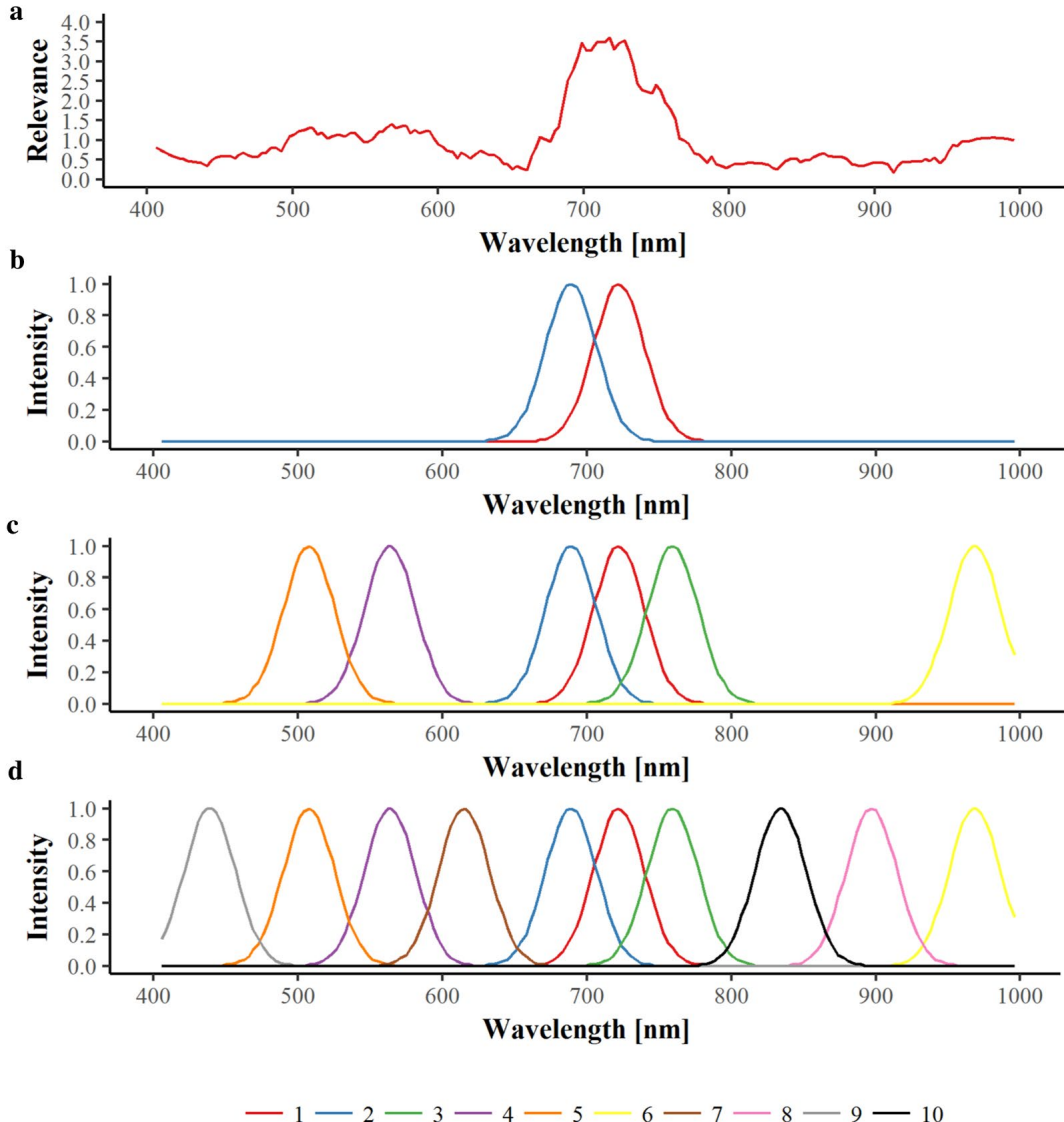
Example of the multispectral simulation. A relevance profile was calculated that indicates the importance of wavelengths for disease detection (**a**). Based on this relevance profile prototypical wavelengths (band width 30 nm) can be placed in important wavelength ranges. Starting with the two most informative bands (**b**), single bands can be added (**c**) according to their importance until a maximum of ten bands (**d**) is reached

In order to improve the efficiency of hyperspectral data analysis and to transfer the detection systems to a more practical application, data dimensionality has to be reduced. Therefore, up to ten local maxima were selected based on the relevance profiles to simulate a multispectral system (see Additional File 2). More than ten wavebands were seen as not realistic for commercial multispectral cameras. A threshold avoids overlapping between selected spectral bands. An example of the band selection methodology is depicted in Fig. 3.

Table 4 shows CAs, TPRs and FPRs for the dimensionality reduction. In addition to results of the two to ten most important spectral bands, the entire spectrum is represented, which consist of 186 and 288 bands in the VNIR and SWIR range, respectively. Furthermore, the wavelengths of the MicaSense RedEdge™ camera used for airborne multispectral data acquisition were selected and simulated. In general, regarding the symptom detection models, the TPRs increased and the FPRs decreased as more wavelengths were considered. Again, the model using annotated data performed better than the model using original field data. For the annotated detection model, the TPRs were usually lower and the FPRs usually higher for the multispectral simulation in comparison to the complete spectra. Regarding the model based on original field data, the TPRs of the best performing multispectral approach were always higher than those of the entire spectrum. For the pre-symptomatic model, the FPRs decreased, but the TPRs did not necessarily increase when considering more spectral bands. The RedEdge™ wavelengths showed satisfying results but performed worse than the entire spectrum and best band selection approach, which is indicated by higher FPRs and lower TPRs.

**Table 4:** Results of the multispectral simulation for the three differentiation tasks

			VNIR												SWIR								
			HSI	Multispectral										HSI	Multispectral								
			186	2	3	4	5	6	7	8	9	10	RE	288	2	3	4	5	6	7	8	9	10
2016	Symptomatic (original)	CA (%)	73	60	66	71	72	74	75	75	76	76	70	73	58	60	63	65	66	67	68	69	70
		TPR (%)	71	77	71	70	72	72	79	77	77	78	71	70	66	66	66	65	69	66	73	71	74
		FPR (%)	29	37	39	27	26	25	26	26	24	24	30	34	50	40	35	35	34	35	33	30	32
	Symptomatic (annotated)	CA (%)	92	68	69	79	84	86	91	90	91	91	82	88	61	85	85	86	86	86	87	87	87
		TPR (%)	89	69	72	78	83	86	88	88	88	88	86	86	84	71	71	75	76	77	79	81	81
		FPR (%)	0	32	31	19	13	10	6	6	7	6	22	2	48	1	1	3	3	6	4	7	6
	Pre-symptomatic	CA (%)	–	–	–	–	–	–	–	–	–	–	–	–	–	–	–	–	–	–	–	–	–
		TPR (%)	–	–	–	–	–	–	–	–	–	–	–	–	–	–	–	–	–	–	–	–	–
		FPR (%)	–	–	–	–	–	–	–	–	–	–	–	–	–	–	–	–	–	–	–	–	–
2017	Symptomatic (original)	CA (%)	70	57	61	66	70	70	71	72	72	73	68	81	62	66	71	74	78	78	79	81	83
		TPR (%)	73	80	68	74	74	72	73	78	76	77	72	82	81	77	75	80	84	82	81	84	86
		FPR (%)	32	45	42	39	33	32	32	31	30	30	35	26	44	43	34	31	27	27	23	22	18
	Symptomatic (annotated)	CA (%)	90	67	77	73	85	85	86	87	86	89	83	95	65	66	71	77	79	84	87	88	89
		TPR (%)	90	74	76	81	87	89	89	89	88	91	84	90	77	77	79	78	78	86	86	89	87
		FPR (%)	11	41	22	34	16	17	15	14	15	13	19	6	44	33	31	24	22	16	12	11	11
	Pre-symptomatic	CA (%)	62	53	54	56	59	60	61	62	63	65	59	74	57	62	65	66	67	68	70	71	71
		TPR (%)	63	38	54	48	61	65	66	60	56	64	56	78	68	71	71	70	77	73	81	77	77
		FPR (%)	35	36	39	36	44	36	32	33	34	31	37	32	52	45	40	41	39	36	36	35	36
2018	Symptomatic (original)	CA (%)	77	63	63	73	75	75	76	76	77	77	68	80	60	60	68	70	72	75	75	77	79
		TPR (%)	72	74	66	73	76	74	74	77	77	80	67	74	56	60	64	67	68	74	78	78	82
		FPR (%)	22	41	33	35	25	22	23	25	24	23	31	20	41	37	27	30	28	23	25	21	20
	Symptomatic (annotated)	CA (%)	94	71	80	81	82	83	89	90	91	91	84	92	67	73	76	79	85	87	87	88	89
		TPR (%)	93	71	81	82	82	85	89	89	91	90	83	100	68	65	73	72	83	84	86	87	88
		FPR (%)	5	25	21	20	17	16	11	10	10	10	16	5	28	17	17	16	12	10	12	10	9
	Pre-symptomatic	CA (%)	79	62	66	68	70	71	74	74	75	76	69	86	60	63	65	75	77	78	80	81	82
		TPR (%)	83	77	73	78	82	82	78	80	81	80	72	87	54	71	63	78	80	80	82	84	79
		FPR (%)	28	41	35	39	24	35	31	31	29	29	34	21	42	40	42	24	22	22	19	17	19

*CA*  classification accuracy, *TPR *  true-positive rate, *FPR *  false-positive rate, *HSI * Hyperspectral Imaging, *RE * simulation RedEdge™ wavelengths, 186/288 = wavelength bands of the hyperspectral sensors, 2–10 = number of selected spectral bands

### Airborne multispectral detection of Esca symptoms

In the multispectral simulation approach, the RedEdge™ wavelengths were simulated and showed acceptable CAs meaning that the camera in general is capable of detecting foliar Esca symptoms. This multispectral camera was then used in an airborne disease detection approach, which was conducted according to vines’ disease severity. Due to the small amount of vines with < 25% and > 75% symptomatic leaves, only results for plants with 25 to 75% symptomatic leaves are listed in Table 5. CAs between 58 and 73% for both years and classes were achieved, indicating that airborne symptom detection is a challenging task, as expected. The FPRs were similar between disease severity classes per year, but the TPRs varied. For disease severity class 2, 57 and 45% of all symptomatic vines were detected in 2017 and 2018, respectively. Regarding disease severity class 3, TPRs of 60 and 74% could be shown, implying a slight trend towards better detection of Esca symptoms with higher disease severity.

**Table 5:** Results for the multispectral detection of Esca leaf symptoms

		2017	2018
Disease severity class 2 (25–50% symptomatic)	CA (%)	60	58
	TPR (%)	57	45
	FPR (%)	36	28
Disease severity class 3 (50–75% symptomatic)	CA (%)	65	73
	TPR (%)	60	74
	FPR (%)	30	28

*CA * classification accuracy, *TPR* true-positive rate, *FPR*  false-positive rate

## Discussion

Disease detection was performed in a vineyard heavily affected by Esca using hyperspectral as well as multispectral imaging. To the authors’ knowledge, this is the first in-field approach over three consecutive years concerning the detection of the Esca disease. Detection of leaf symptoms was performed using original field data as well as annotated data. In general, symptoms appear only on single leaves or shoots and do usually not affect the whole plant [37]. Field data is therefore a mixture of symptomatic and non-symptomatic leaves, which may influence the performance of a disease detection model. By using annotated data, not the entire plant, but only symptomatic leaves were considered in model development in order to simulate the best possible conditions. The results show that the model using annotated data performed significantly better with high TPRs in the range of 86—100% and low FPRs, in contrast to the model using original field data. However, when applied on plant level, the model using original field data could detect more symptomatic plants. This model was trained on heterogeneous field data and can therefore be considered more robust as well as better adjusted to mixed leaf samples than the model trained on optimized annotated data. A comparable approach using RGB-images was performed by Rançon et al. [33] who, at first, tested the feasibility of Esca symptom detection on selected leaves, thereby, achieving satisfying overall accuracies of 88 and 91% for white and red cultivars, respectively. In a next step, they evaluated disease detection on plant-scale and found it to be more complex due to varying symptom intensity and overlapping effects of leaves.

FPRs can be adjusted in the model application, thereby, influencing TPRs and CAs. Setting a FPR threshold depends on a compromise between the detection of all symptomatic plants and no misclassification of healthy plants. In this study, the FPR threshold was set to obtain the best TPRs with the consequence of lower CAs. This idea is in line with Qin et al. [38] who developed a detection model for cankerous grapefruits and decided the economic losses of missing infected fruits to be much higher than the false classification of a healthy fruit. Thus, for practical application using a disease detection system is only the first step in disease management. As the next step winegrowers need to visit detected plants in the field and decide on appropriate measures. Therefore, it is easier to notice that one plant was misclassified than to omit a symptomatic one.

The transferability of the two-year models on the third experimental year showed very divergent results depending strongly on the year and wavelength range. In general, VNIR seems to be more stable. Reflectance of leaves in the VIS region is mainly affected by the absorption of photosynthetic pigments and in the NIR by internal structures [39]. Development of foliar Esca symptoms leads to drastic alterations of the photosynthetic apparatus and strongly affects cell structure [5, 40], which is expressed in chlorotic and necrotic tissue. In contrast, reflectance in the SWIR range is influenced by the composition of leaf chemicals and water [11] and could therefore display not only the impact of Esca but also of other metabolomic variations due to environmental factors. The model 2017/18 performed worst when applied on the third year (2016) in both the VNIR and SWIR range, which could potentially be explained by the different camera systems used. In 2016, another camera system was used as in 2017 and 2018. Although, measurements were performed in the same wavelength ranges and camera to camera transformation was realized by nearest wavelength interpolation scheme, the influence of the two different camera systems on experimental results cannot be fully excluded. Another possible explanation is the weather conditions in the 3 years. While 2016 was moderate in temperature and very rainy, the weather in 2017 and 2018 was rather hot and dry leading to different annual and environmental conditions (and stresses) for the plants. It is generally assumed that environmental factors influence the disease and thus symptom development (for review see [41]). Hence, a disease detection model developed on 2 years seems not sufficient for the consistent prediction of Esca symptoms in the field. Further experimental years should be added to soften annual fluctuations. In this pilot study, Esca disease detection was only performed on one white grapevine cultivar and should therefore also be applied to others, since there is a large diversity of symptoms between red and white varieties [37]. This will be another challenging task in testing the transferability of disease detection models because spectral reflectance differs by cultivar as recently demonstrated by Al-Saddik et al. [42] and Gutiérrez et al. [43].

The pre-symptomatic detection approach showed very promising results, especially in 2018. It could be possible that symptoms in 2018 developed a few days earlier than in 2017 and therefore better results might be obtained. This may be explained by a faster vine development in 2018 than in previous years, as observed by Kraus et al. [44]. Perhaps a shorter period between hyperspectral data acquisition and visual assessment should be chosen to gain further information on changes in spectral reflectance prior to symptom development. The reasons of symptom development still remain elusive. So far, pathogens could not be isolated from leaves of infected plants. Therefore, it is hypothesized that fungal phytotoxins are transported from the wood to the leaves causing morphological and physiological modifications [45, 46]. Esca greatly affects primary and secondary metabolism in pre-symptomatic leaves leading to alterations in the composition of leaf biochemicals and subsequently causing changes in reflectance spectra [9, 47]. These alterations as well as the absence of visual symptoms make the SWIR range the better predictor of grapevine’s disease status for the early detection. The complexity of the Esca disease has hindered the development of efficient control strategies. An early detection system could therefore not only contribute to the understanding of symptom development but also help to reduce disease spread.

In this study, hyperspectral imaging was used as the basis for the identification of application specific wavelengths. Thereby, reduced data dimensionality enables a more efficient computational analysis and processing. Based on these wavelengths an inexpensive multispectral sensor could be designed that would allow for faster data acquisition while being mounted on a tractor or UAV. Identification of optimal spectral bands is a common approach that has successfully been implemented for the detection of three sugar beet diseases [13], yellow rust on winter wheat [48], anthracnose on strawberries [49], or Flavescence dorée on grapevine [42]. Yeh et al. [49] and Moshou et al. [48] found that the number of selected wavelengths significantly influences CAs. This confirms our results—by using more significant wavelengths, better classification could be achieved. The multispectral simulation performed even better than the whole spectral region regarding original data.

Within this multispectral simulation, spectral bands of the MicaSense RedEdge™ camera were applied on the hyperspectral data. Acceptable results for original field data and satisfying CAs of 82—83% for annotated data could be achieved indicating that symptom detection is possible. This simulation was then compared to genuine multispectral data of the RedEdge™ camera collected by a UAV. In general, using a multispectral sensor attached to a UAV allows for fast and flexible data acquisition with high resolution and low operational costs [50]. Airborne disease detection seems to be challenging with rather low CAs of 58—73%. However, a light trend towards better Esca detection with higher disease severity could be observed. A similar study was performed by Albetis et al. [36] concerning the airborne identification of grapevines affected by GTD and Flavescence dorée. They found differences of predicted and actual disease severity, especially for vines with less than 50% symptomatic leaves. Di Gennaro et al. [35] excluded vines with Esca symptoms less than 20% from their analyses, since they were barely detectable on airborne multispectral images. In principle, the multispectral camera is capable of detecting Esca symptoms as could be seen in the simulation, but showed its limitations within the airborne approach. Modifying the airborne experimental setup might possibly improve classification. The major difference comparing ground-based and airborne data acquisition is the view angle [51]. The Phenoliner collects hyperspectral data in a distance of 1 m and face-on the grapevines, while the UAV flies in a distance of 30—40 m using a nadir view. In the common vertically shoot positioned (VSP) growing system leaves face into the corridors and rarely upwards [52]. Additionally, symptomatic leaves are not necessarily found in the upper canopy. Di Gennaro et al. [53] used a side view of the canopy for the automated estimation of yield. They could detect more than 85% of all clusters in the grape zone and were able to predict yield with more than 84% accuracy. In further experiments using UAV, lower angles could capture vine canopy laterally. This might improve disease detection and subsequently disease management.

## Conclusion

Esca is a grapevine disease of great economic importance for winegrowers, but little is known about the relation of internal and external symptoms. A monitoring system could help understanding the dynamics of symptom development and disease spread. The overall aim of this study was to evaluate spectral sensors for the in-field detection of Esca. Several approaches for disease detection were tested using hyperspectral and multispectral imaging. Ground-based hyperspectral imaging could successfully be used to detect foliar symptoms. However, transferring the disease detection models to unknown data remains a challenging task. Initial tests for the pre-symptomatic detection of infected vines showed promising results, which could also contribute to the knowledge and control of the disease. By selecting disease specific spectral wavelengths, a multispectral approach was simulated and compared to airborne multispectral data. Although, the experimental setup for airborne disease detection needs to be optimized, both sensor systems seem to be suitable for the in-field detection of Esca. In this study, only one grapevine cultivar was investigated. Further research is needed to verify these results. Moreover, Esca symptoms need to be discriminated from other grapevine diseases and deficiencies producing similar changes in leaf coloration.

## Methods

### Field tests

Field tests were conducted from 2016 to 2018 in an experimental vineyard heavily infected with Esca at the JKI Geilweilerhof located in Siebeldingen, Germany (49°21.7470 N, 8°04.6780 E). The vineyard consisted of *Vitis vinifera* L. cv. ‘Phoenix’ planted in 1996. Rows of 120 m length were oriented in east–west direction. Interrow and grapevine spacing was 1.8 and 1.3 m, respectively. Grapevines were trained in a VSP-system and pruned as single guyot. The precise location of all plants was acquired using a portable GPS (SPS585, Trimble®, Sunnyvale, CA, USA) providing highly accurate positioning (resolution: 0.02 m).

The acquisition of spectral data was conducted at several time points during the growing season depending on vine and symptom development to record vines pre-symptomatically and with different disease severity levels. In 2016, vines were recorded at the beginning of August and September; in 2017, in June, July and August; and in 2018, in July and August—always before harvest. The plot was monitored biweekly—in parallel to hyper- and multispectral field tests—and showed an average incidence of chronic Esca leaf symptoms of 11%. Two rows of the experimental vineyard were recorded in all 3 years. This results in 129, 114, and 112 monitored plants for the 3 years, respectively. Due to the persistently high number of Esca symptomatic vines within this vineyard, many vines had to be removed in recent years. In 2017, an additional row with 69 vines was considered.

### Hyperspectral imaging

#### Data acquisition

**Fig. 4:**
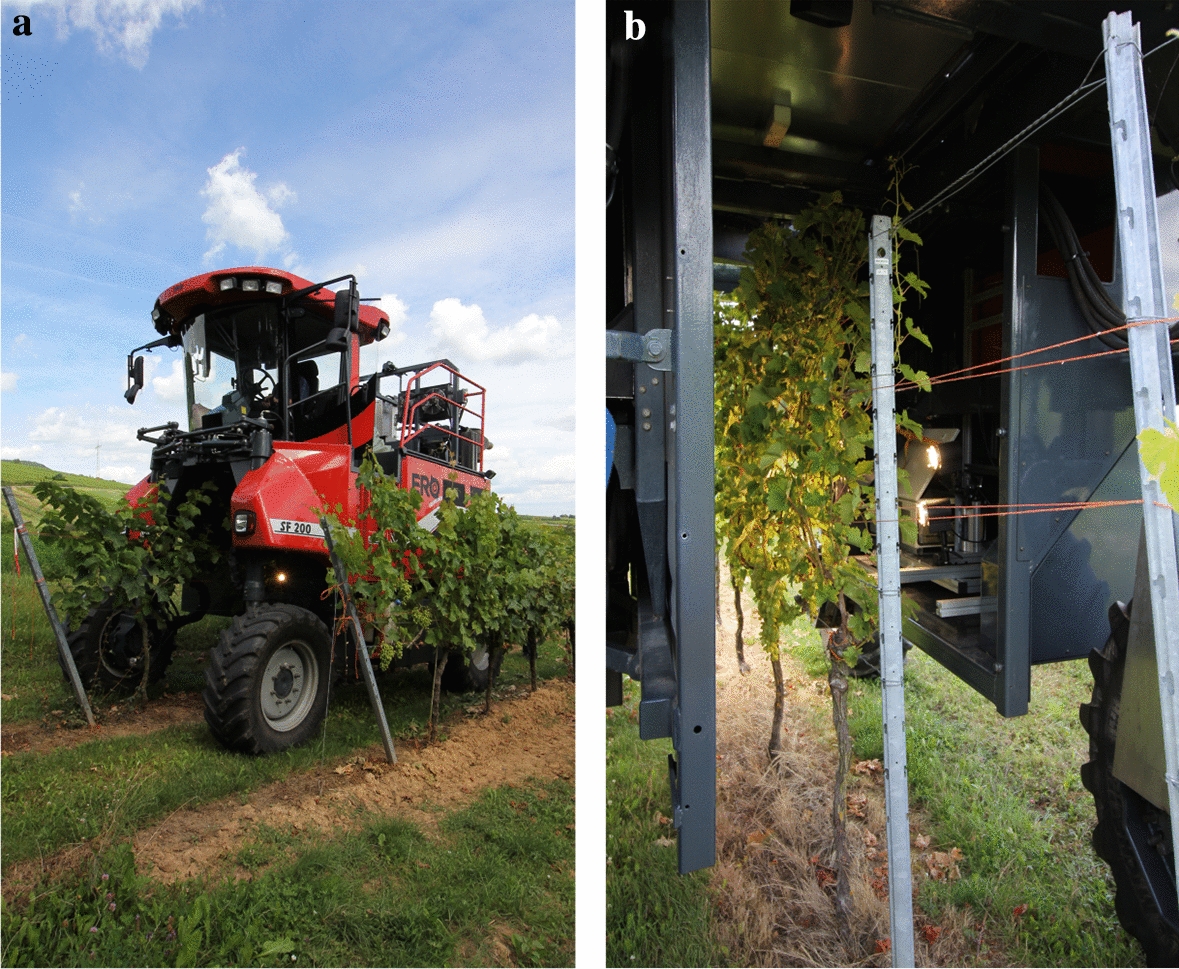
Depicted are the Phenoliner driving through the vine rows (**a**) and a close up view of the tunnel during data acquisition (**b**)

Hyperspectral imaging was performed using the Phenoliner (Fig. 4), a novel field phenotyping platform, which was recently introduced by Kicherer et al. [54]. Due to its tunnel structure, it avoids disturbing environmental factors (e.g. sunlight, effects of clouds) and enables the acquisition of field data under almost standardized conditions. Few modifications have been made in comparison to the original design. First, additional light sources were mounted for a better illumination of the recorded area consisting of two 300 W short-wave spotlights (Hedler C12, Hedler Systemlicht, Runkel/Lahn, Germany) with a broad power spectral density. Second, the PTFE spectralon (1 × 1 m) used as a calibration standard was replaced by a smaller one (54.5 × 35 cm) (Sphere Optics, Herrsching, Germany) covered with a borosilicate glass for protection. The calibration standard was reflectance certified by Sphere Optics GmbH (Herrsching, Germany).

Spectral reflectance range from 400 to 2500 nm was measured with two separate line scanning cameras (HySpex®) supplied by Norsk Elektro Optikk A/S, Skedsmokorset, Norway. During the course of the project, two sets of cameras were used. Spectra in the visible and near-infrared range (VNIR; 400–1000 nm) were recorded in 2016 by a HySpex VNIR 1600 and in 2017 as well as in 2018 with a HySpex VNIR 1800. The spectral acquisition in the short-wave infrared (SWIR; 1000–2500 nm) was performed in 2016 with a HySpex SWIR 320 m-e and in 2017 as well as in 2018 with a HySpex SWIR 384. In Table 6 an overview of the camera specifications is given.

**Table 6:** Key specifications of the camera systems used

Specification	HySpex VNIR 1600	HySpex VNIR 1800	HySpex SWIR 320 m-e	HySpex SWIR 384
Dynamic range (bit)	12	16	14	16
Spectral bands	160	186	256	288
Spatial pixel	1600	1800	320	384
Max. framerate (Hz)	100	100	160	400
Spectral resolution (nm)	3.70	3.26	6.00	5.45

Spectral camera to camera transformation was achieved by nearest wavelength interpolation scheme. As a calibration standard, the PTFE spectralon is recorded constantly in the background of the camera viewport. For the correct mapping of spectral data and vine position, a proprietary image acquisition software developed by Fraunhofer Institute for Factory Operation and Automation IFF was used, which integrates the two hyperspectral sensors and the Phenoliner GPS.

#### Data calibration and labeling

Data calibration was performed by calculating the reflectance value from a recorded dark current (while camera shutter is closed) and a recording when the spectralon (white target) is covering the camera viewport. Calibration measurement is performed before and after each grape row recording. Reflectance ${R}_{\lambda }$ per pixel is calculated as

$${R}_{\lambda }=\frac{{I}_{\lambda }-{I}_{\lambda }^{DC}}{{I}_{\lambda }^{W}-{I}_{\lambda }^{DC}}$$

where ${I}_{\lambda }$ is the image pixel intensity at wavelength $\lambda$, ${I}_{\lambda }^{DC}$ the intensity when measured with closed shutter (“dark current”) and ${I}_{\lambda }^{W}$ being the intensity while recording the spectralon device. Based on the reflectance image, a classification model was trained to classify each spectral pixel into either vegetation, grapes, stems and general background. The classification model training was performed with the AutoML platform HawkSpex®Flow developed by the Fraunhofer IFF. The best combination of model and background was selected and used for all segmentation purposes. The winning model was a Multi-Layer Perceptron [55] with SNV normalization [56]. Separate models for the VNIR and SWIR image processing were created. For all further analyses only pixels classified as vegetation were used; all other pixels (grapes, stems, and background) were discarded.

In order to label leaf material with visual rating data, two different approaches were followed:

##### Vine based GPS labeling

While recording the hyperspectral images, a log with GPS positions was created. GPS positions were corrected for the relative position of GPS antenna and camera position within the vehicle. From a list of manually measured vine locations, a vine ID was associated with an image window centered on the scanning line with the closest distance to the vine GPS position. The window was set to 50 cm in order to avoid overlapping of vine plans. The window was further adjusted by considering the vines arc direction. Labels for symptomatic rating as well as pre-symptomatic rating were associated to the data via the vine ID. Pre-symptomatic plants did not show symptoms at the moment of hyperspectral imaging but within the next two weeks.

##### Manual labeling of Esca symptoms

Field data is a heterogeneous mixture of symptomatic and non-symptomatic leaves; this could potentially influence the performance of a disease detection model. In order to acquire precise ground truth data and simulate best possible conditions, RGB images were reconstructed from the VNIR and SWIR camera separately. Reconstruction was performed using three wavelength reflectance values as RGB values. Esca symptoms were manually labeled using the Paint.NET software. As control vegetation, data from vine labeled as uninfected were extracted using the approach in a). Since pre-symptomatic vines did not show any symptoms at the time of recording, they were not labeled manually. After an annotated labeling mask was available, this mask was used to sample the data. This generated the positive reflectance samples that were combined with negative samples from control vines to form a new dataset for the modelling experiments. The mask was generated on the VNIR image and projected on the SWIR image to generate samples from both wavelength ranges. All processing was done by the Fraunhofer IFF HawkSpex®Flow software, which is implemented in Matlab (Matheworks Inc.).

#### Detection model development

The labeled data formed a number of datasets for the subsequent machine learning for creating a detection model. A machine learning model is a generic mathematical formula that calculates an output from an input using a number of free parameters. Best-known methods are Artificial Neural Networks, which mimic the information processing of the human brain, or method motivated from statistics like Support Vector Machines (SVM) or Bayesian that model data distributions. For each method, an algorithm is defined that finds the optimal parameter of the model by calculating the error between the model output and a target value. In this case, a coded representation of the visual rating class associated with the data point. Coding of −1 for control and + 1 for pathogen infection was used (either symptomatic or pre-symptomatic). The optimization algorithm then finds parameters for the model that minimizes the error, either by iteratively applying an optimization strategy like gradient descent (Artificial Neural Networks) or by a closed form mathematical calculation (e.g. Partially Least Squares).

An important aspect of data-driven modeling is the cross-validation scheme. In this study, an n-fold cross validation with n = 10 is performed with the dataset being divided into n parts. The model is the one optimized on n-1 folds while tested on the n^th^-fold. The modeling process is performed with all possible combinations without repetition of folds. As a model performance indicator, the average and standard deviations of the performance value is calculated across modeling runs.

All models where assessed using the following performance criteria:

- *True Positive Rate (TPR):* $$TPR=\frac{number of samples detected correctly as Esca infected}{total number of control samples}$$
- *False Positive Rate (FPR):* $$FPR=\frac{number of samples detected incorrectly as Esca infected}{total number of control samples}$$
- *Overall Accuracy (CA):* $$CA=\frac{total number of correctly detected samples}{total number of samples}$$

For the model development, one sample is constituted by a pixel spectrum; while for application of the model with majority vote, the whole plant constitutes one sample. A number of spectral preprocessing methods in combination with machine learning models were tested (Tables 7 and 8):

**Table 7:** Pre-processing methods

Method	Formula
Vector L2 normalization	${R}_{\lambda }^{N}=\frac{{R}_{\lambda }}{\sqrt{\sum_{\lambda }{{R}_{\lambda }}^{2}}}$
Vector SNV normalization [56]	${R}_{\lambda }^{N}=\frac{{R}_{\lambda }-\frac{1}{N}\sum_{\lambda }{R}_{\lambda }}{\sqrt{\frac{1}{N-1}\sum_{\lambda }{\left({R}_{\lambda }-\frac{1}{N}\sum_{\lambda }{R}_{\lambda }\right)}^{2}}}$

**Table 8:** Model hyper-parameters

Method	Citation	Hyper-Parameters
Multi-layer perceptron network (MLP)	[55, 57]	Number of hidden layers: 3 Optimization method: scaled conjugate gradient backpropagation, learning rate self-adapting Neurons per hidden layer: 50, 25, 10 Loss function: mean-squared error
Radial-basis function network with relevance (rRBF)	[58–60]	Number of radial basis functions: 30 Optimization method: scaled non-linear conjugate gradient, learning rate self adapting Loss function: mean-squared error
Partially least square (PLS)	[61]	Number of components: 20
Linear discriminance model (LDA)	[62]	No hyper-parameters

The models were generated on datasets describing a certain detection task with data separately for the measurement years 2016, 2017, and 2018 as well as combined datasets for 2 years in all possible combinations. These 2 year models where then used to detect samples from the third year in order to test their transferability to an independent year. Due to computational demand, only 10,000 spectra per label class were used in the modeling. VNIR und SWIR reflectance data is modeled separately since an alignment of camera images was not successful, partly due to different camera resolutions.

#### Application of detection model

**Fig. 5:**
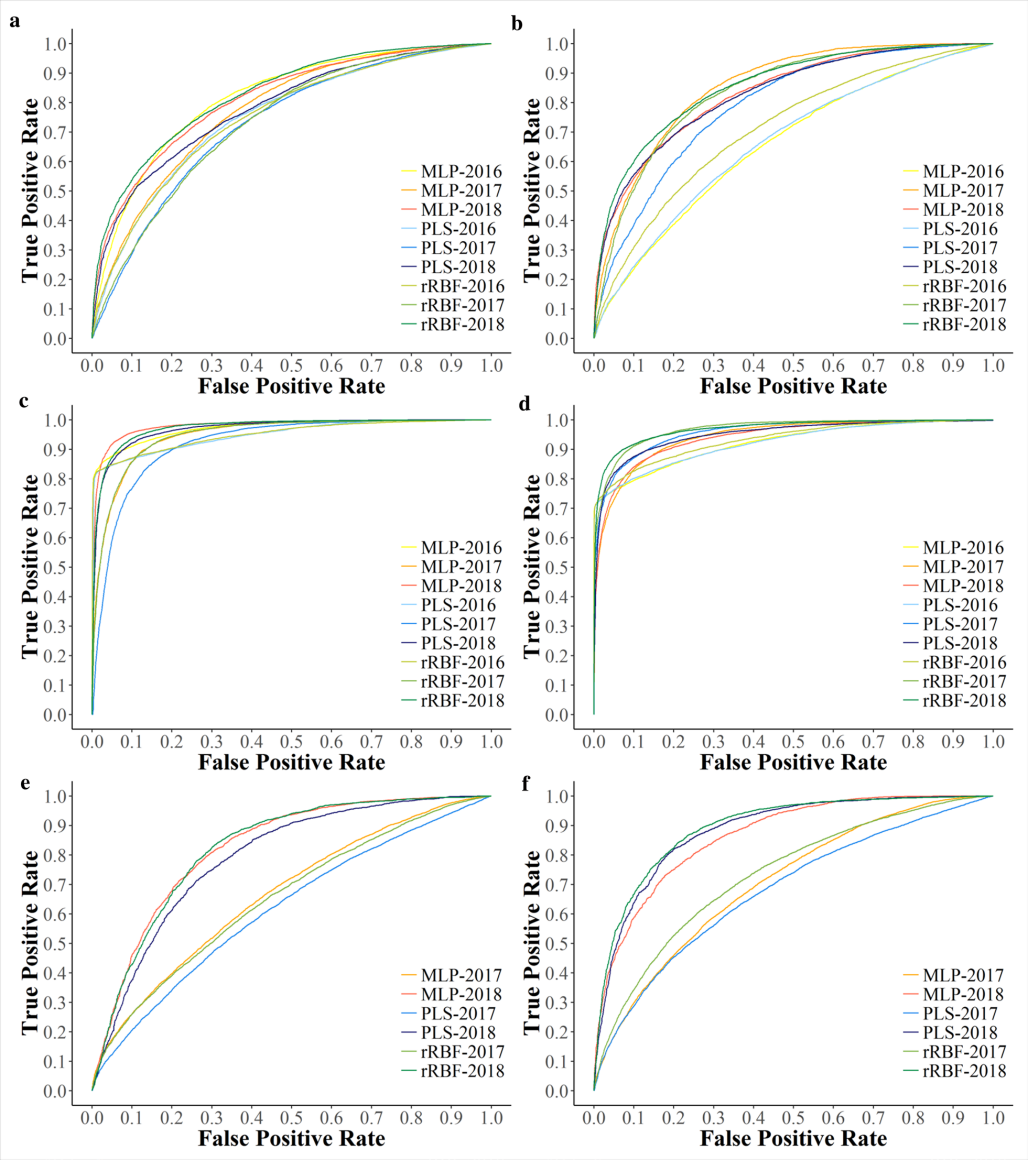
ROC curves of Esca disease detection in 2016, 2017, and 2018 using PLS, MLP, and rRBF, respectively. Curves are depicted for original data (**a**, **b**), manually annotated data (**c**, **d**), and pre-symptomatic data (**e**, **f**) using the VNIR (**a**, **c**, **e**) and SWIR (**b**, **d**, **f**) range, respectively

Once optimized to the detection task, the best performing model in terms of accuracy (see Additional Files 3 and 4) was then applied back to the hyperspectral image. In most cases, MLP was the best performing model for VNIR data and rRBF for SWIR data. For better visual presentation, receiver operator characteristics (ROC) are depicted in Fig. 5. ROC curves are based on TPR (or sensitivity) and FPR (or 1-specificity).

Each vegetation pixel was labeled using the detector. In order to assess the detection performance of the detector a statistics over all vegetation pixels within the vine window as described above was calculated and the label with the highest occurrence was deemed as the representing label for the whole vine (majority vote).

**Fig. 6:**
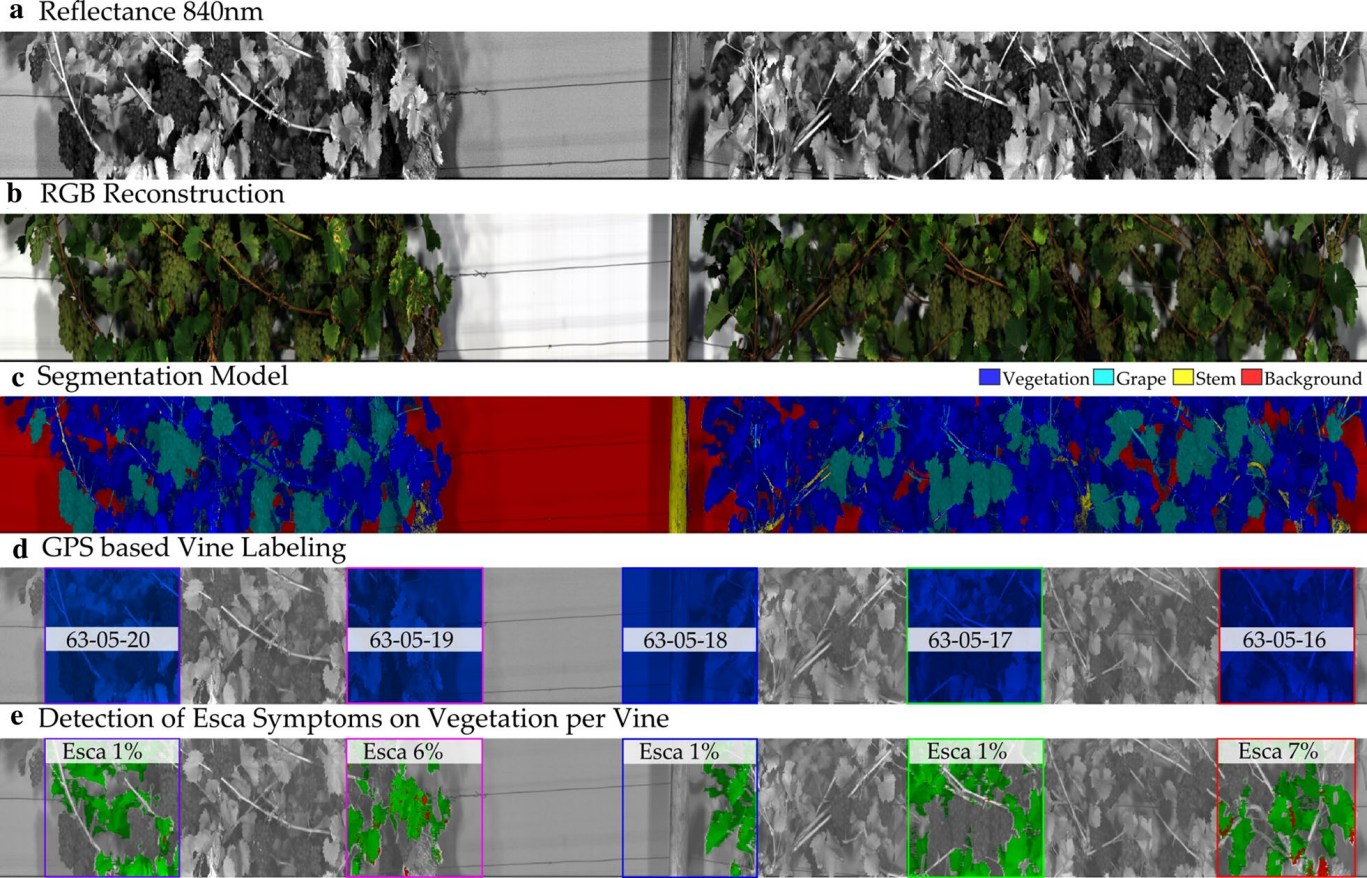
Hyperspectral imaging steps. A calibrated reflectance image (**a**) is used to reconstruct a RGB-image (**b**) for manual annotation. The reflectance image (**a**) is segmented into its main components (**c**) and vines are labeled based on their GPS position (**d**). The disease detection model is then applied to the pixels associated with the GPS vine position and the detection ratio is calculated (**e**)

In Fig. 6 the hyperspectral imaging steps are shown, a calibrated reflectance image (a) is semantically segmented for its principal material components (c) and labeled for vine positions from GPS (d). A detector is applied to each pixel and the ratio detection decision within a vine window is calculated (e).

#### Spectral relevance and multispectral simulation

The multispectral simulation approach is basically a feature selection problem, where in a set of high-dimensional input features only a subset of relevant features ought to be used to reduce the input variables. Hyperspectral data consists of a continuously sampled reflectance spectrum in a certain wavelength range, so, neighboring features are highly correlated to each other and constituted a “functional” feature vector.

In order to place a certain number of multispectral channels, a weighting profile λ over the wavelength range is necessary. This weighting profile (also called relevance profile) is acquired in this study by weighting the Euclidian distance in the Radial Basis Function Network with Relevance (rRBF) [59] with loss function $E$:

$$E\left(X,W,\uplambda \right)=\frac{1}{2}\sum_{r}\sum_{k}{\left({y}_{k}\left({x}_{r}\right)-{t}_{k}^{r}\right)}^{2}$$

$${y}_{k}\left({x}_{r}\right)=\sum_{n}{u}_{nk}\varphi \left(d\left({v}_{r},{w}_{n},\lambda \right)\right)$$

$$d\left({x}_{r},{w}_{n},\lambda \right)=\sum_{b}{\lambda }_{b}{\left({x}_{rb}-{w}_{bn}\right)}^{2}$$

where ${x}_{r}$ is the input reflectance, ${t}_{k}^{r}$ the class coding (one-hot coding), $\varphi$ a Gaussian function and $d(\bullet )$ the Euclidean distance between the prototype ${w}_{n}$ and the input reflectance ${x}_{r}.$ By optimizing $E$ for parameters in $W$ and $\uplambda$, the weighting parameter is adjusted so it amplifies relevant input channels while suppressing irrelevant features. The weighting profile is forced to a sum of 1 therefore generating some sort of winner-takes-all competition between features.

When generated, the weighting profile is used as a probability distribution function (pdf) and a number of 10,000 random samples are generated according to this pdf. In that way, in areas of high relevance, samples are denser then in areas with low relevance. This dataset can now be used with a vector quantization algorithm like Neural Gas to place prototypical wavelengths. The Neural Gas was used as described here [63]. In order to simulate a multi-spectral response, one has to take into account that a multi-spectral camera does not offer the same narrow band sampling like a hyperspectral camera. Therefore, a band of 30 nm full-width-half-maximum (FWHM) is assumed which is typical for multispectral cameras. The simulated response of a multispectral camera is then calculated by

$${r}_{rm}^{MSI}=\frac{1}{{\sum }_{b}{g}_{m}({\alpha }_{b})}\sum_{b}{x}_{rb}{g}_{m}\left({\alpha }_{b}\right)$$

which basically multiplies a Gauss kernel representing the spectral channel at a center wavelength ${\mu }_{m}$ and of sigma $\sigma$ with the reflectance spectrum.

$${g}_{m}\left({\alpha }_{b}\right)=\frac{1}{\sigma \sqrt{2\pi }}\mathrm{exp}\left(-0.5{\left(\frac{{\alpha }_{b}-{\mu }_{m}}{\sigma }\right)}^{2}\right)$$

$$\sigma =\frac{FWHM}{2\sqrt{2ln2}}$$

The center wavelength is determined from the Neural Gas prototypes as described above. The simulation is performed for a set of 2 to 10 multispectral channels and modelling is performed on the new input data ${{\varvec{r}}}^{MSI}$ with identical methods as for the hyperspectral dataset.

### Airborne multispectral imaging

Multispectral data was acquired by Lilienthal Digitaler-Weinbau GmbH (Wiesbaden, Germany) during the growing seasons of 2017 and 2018. The whole plot was recorded, but for the analysis only selected rows were considered (see Hyperspectral Data Acquisition).

Multispectral images were collected using a UAV platform (Geo XR6; hexacopter) able to fly by remote control for starts and landings as well as autonomously during image acquisition. Flight altitude was set at 35–45 m above ground with a flight speed of approx. 5 m/s. In this study, the MicaSense RedEdge™ camera (MicaSense Inc., Simi Valley, CA, USA) was used capturing five discrete wavelength bands: Blue (455–495 nm), Green (540–580 nm), Red (658–678 nm), Red-Edge (707–727 nm), and Near Infrared (NIR; 800–880 nm). The multispectral sensor was equipped with an integrated GPS system and a downwelling light sensor (DLS) for better calibration. Using a focal length of 5.5 nm and a field of view of 47.2° the MicaSense RedEdge™ allows the acquisition of 12 Bit RAW data with a resolution of 2.5–3 cm/pixel.

Specific flight patterns were flown to enable an optimal photogrammetric processing. Thus, an automatic soil extraction could be processed to hide information from intra-row vegetation. Data orthorectification was conducted using Agisoft Software (Agisoft LLC., St. Petersburg, Russia). For the analysis of the spectral data, only an area of 50 cm around the GPS vine position (depending on the guyot pruning) is considered. Plants were grouped according to their disease severity (no symptoms, < 25%, 25–50%, 50–75%, and > 75% symptomatic) for data analysis.

The reflectance data in the five camera channels added by the NDVI and NDRE calculated with the following formula served as input to the modeling processing using the same setup as described above for the hyperspectral data processing.

$$NDVI=\frac{{R}_{NIR}-{\mathrm{R}}_{Red}}{{R}_{NIR}+{\mathrm{R}}_{Red}}$$

$$NDRE=\frac{{R}_{NIR}-{\mathrm{R}}_{Red-Edge}}{{R}_{NIR}+{\mathrm{R}}_{Red-Edge}}$$

## Supplementary information

**Additional file 1: Figure S1–S3.** Mean spectra of the different disease detection approaches.

**Additional file 2:** **Table S1.** The ten most informative spectral bands for the differentiation tasks.

**Additional file 3:** **Table S2.** Results of the different machine learning approaches for the 1 year disease detection approaches.

**Additional file 4: Table S3.** Results of the different machine learning approaches for the model transferability evaluation.

## Data Availability

Upon request.
