# Supplementary material for: Evaluating the suitability of hyper- and multispectral imaging to detect foliar symptoms of the grapevine trunk disease Esca in vineyards
Source: Plant Methods. 2020 Oct 21;16:142. doi: 10.1186/s13007-020-00685-3 (PMC7579826; doi:10.1186/s13007-020-00685-3)
Supplement: Supplementary file 1 — Additional file 1: Figure S1–S3. Mean spectra of the different disease detection approaches. [file 13007_2020_685_MOESM1_ESM.docx]

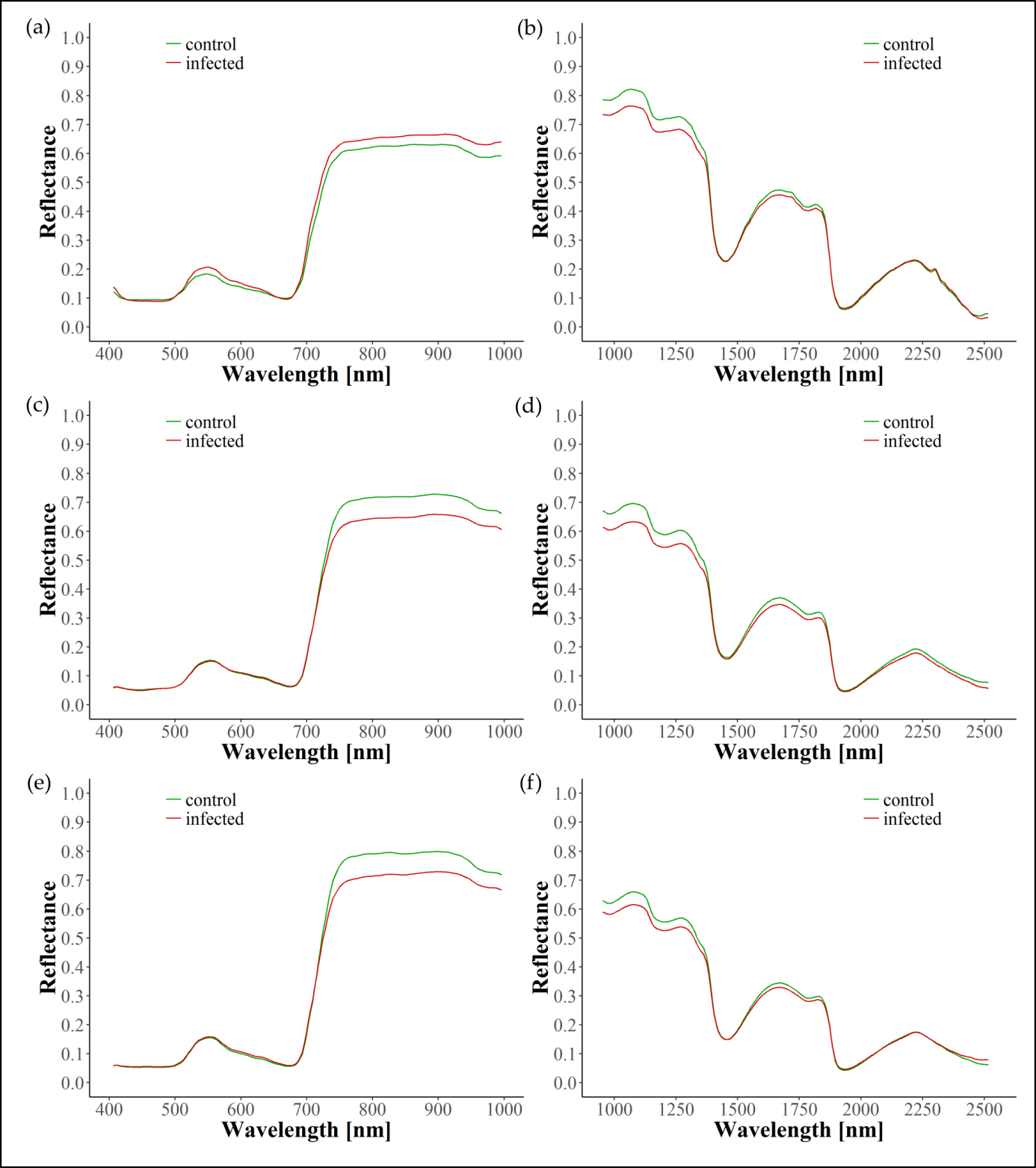


**Figure 1:** Spectral reflectance corresponding to the average of control (green) and infected (red) symptomatic (original) leaves for VNIR (left) and SWIR (right). Spectra are depicted for the years 2016 (a, b), 2017 (c, d), and 2018 (e, f).


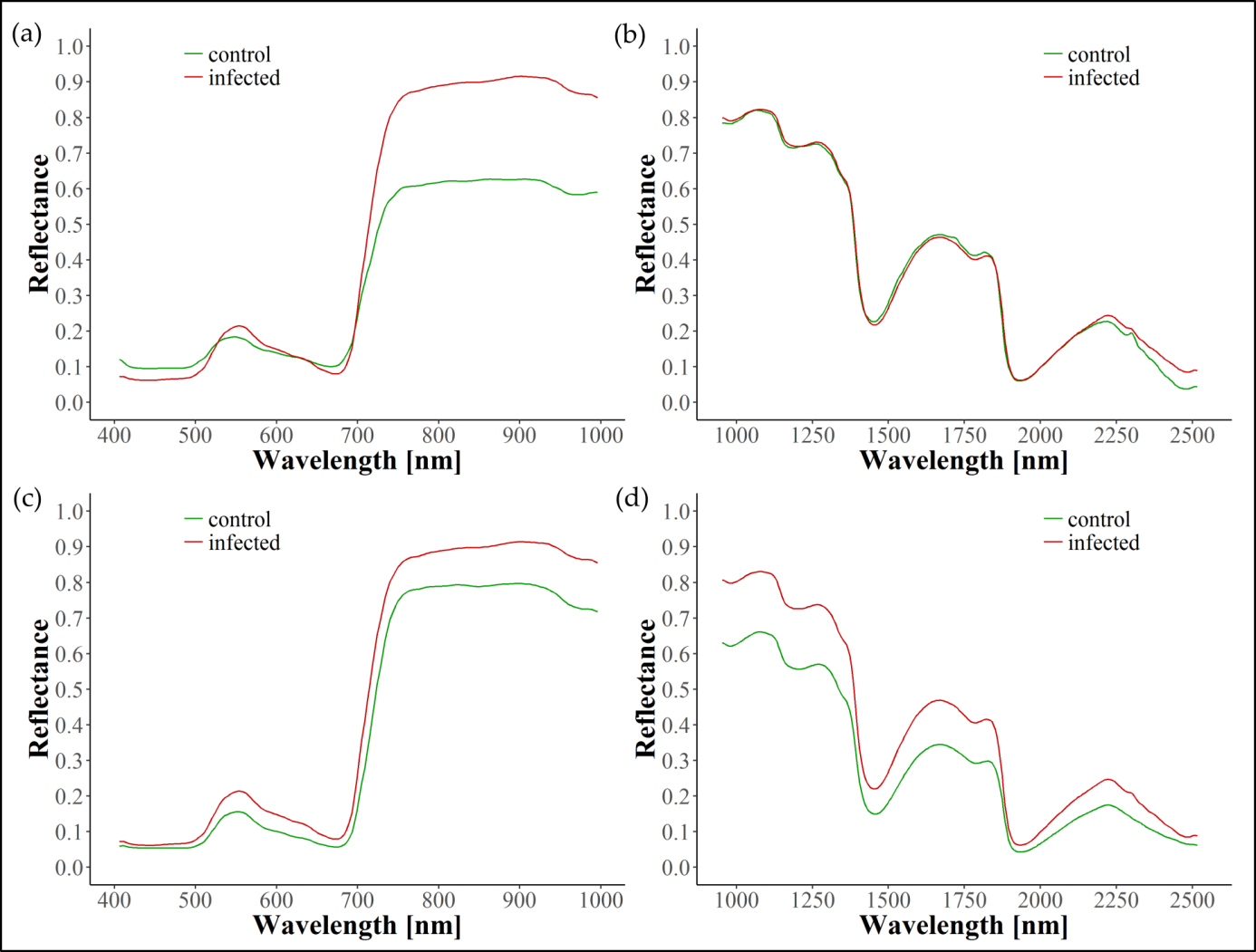


**Figure 2:** Spectral reflectance corresponding to the average of control (green) and infected (red) symptomatic (annotated) leaves for VNIR (left) and SWIR (right). Spectra are depicted for the years 2016 (a, b) and 2018 (c, d).


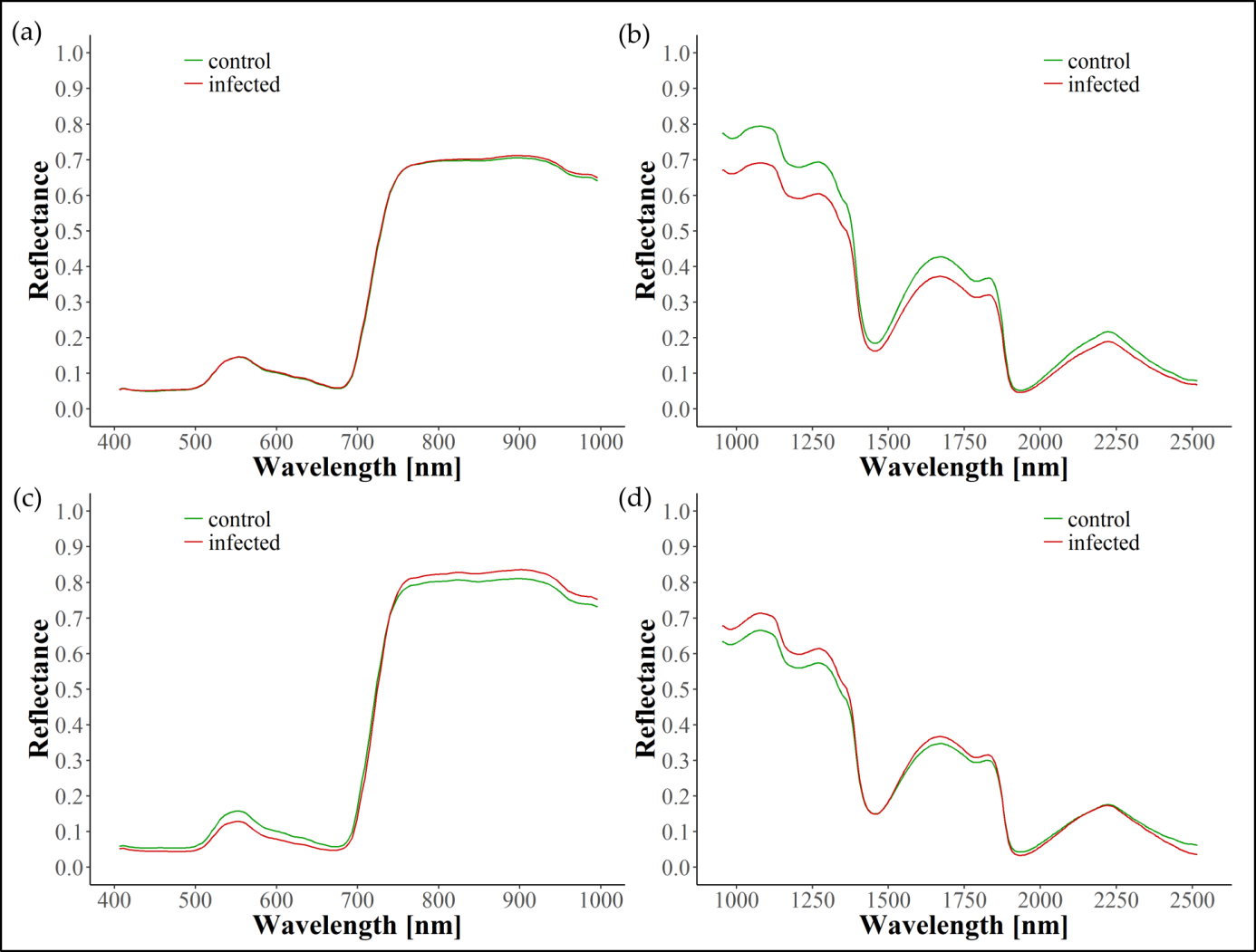


**Figure 3:** Spectral reflectance corresponding to the average of control (green) and infected (red) pre-symptomatic leaves for VNIR (left) and SWIR (right). Spectra are depicted for the years 2017 (a, b) and 2018 (c, d).
