# Supplementary material for: Evaluating the suitability of hyper- and multispectral imaging to detect foliar symptoms of the grapevine trunk disease Esca in vineyards
Source: Plant Methods. 2020 Oct 21;16:142. doi: 10.1186/s13007-020-00685-3 (PMC7579826; doi:10.1186/s13007-020-00685-3)
Supplement: Supplementary file 2 — Additional file 2: Table S1. The ten most informative spectral bands for the differentiation tasks. [file 13007_2020_685_MOESM2_ESM.docx]

Additional Table 1: The ten most informative spectral bands for the differentiation tasks.

|  |  | **VNIR** | | | | | | | | | | **SWIR** | | | | | | | | | |
| --- | --- | --- | --- | --- | --- | --- | --- | --- | --- | --- | --- | --- | --- | --- | --- | --- | --- | --- | --- | --- | --- |
|  |  | **1** | **2** | **3** | **4** | **5** | **6** | **7** | **8** | **9** | **10** | **1** | **2** | **3** | **4** | **5** | **6** | **7** | **8** | **9** | **10** |
| 2016 | Symptomatic (original) | 717 | 556 | 767 | 666 | 497 | 969 | 442 | 613 | 906 | 836 | 1512 | 1708 | 2476 | 2165 | 1368 | 2007 | 1844 | 2313 | 1177 | 1032 |
|  | Symptomatic (annotated) | 722 | 689 | 759 | 564 | 508 | 968 | 615 | 897 | 439 | 834 | 1875 | 1386 | 1518 | 1693 | 2180 | 2338 | 1043 | 2034 | 1212 | 2457 |
|  | Pre-symptomatic | - | - | - | - | - | - | - | - | - | - | - | - | - | - | - | - | - | - | - | - |
| 2017 | Symptomatic (original) | 723 | 973 | 879 | 775 | 537 | 925 | 835 | 674 | 591 | 441 | 1132 | 987 | 2461 | 1724 | 1481 | 1586 | 2080 | 1936 | 2247 | 1317 |
|  | Symptomatic (annotated) | 762 | 477 | 869 | 436 | 815 | 715 | 923 | 662 | 976 | 556 | 1419 | 1004 | 1902 | 1139 | 2087 | 1568 | 1735 | 1280 | 2267 | 2437 |
|  | Pre-symptomatic | 458 | 428 | 948 | 763 | 590 | 808 | 702 | 531 | 651 | 873 | 1147 | 2435 | 1328 | 2116 | 2275 | 1764 | 1647 | 999 | 1464 | 1933 |
| 2018 | Symptomatic (original) | 745 | 781 | 667 | 522 | 913 | 483 | 862 | 429 | 588 | 966 | 1090 | 2230 | 1867 | 1205 | 1496 | 2038 | 1335 | 2349 | 2455 | 1682 |
|  | Symptomatic (annotated) | 750 | 501 | 680 | 789 | 439 | 561 | 900 | 841 | 621 | 958 | 1502 | 1368 | 1863 | 1039 | 2116 | 2447 | 1198 | 1999 | 1695 | 2288 |
|  | Pre-symptomatic | 680 | 557 | 737 | 499 | 785 | 842 | 635 | 901 | 436 | 962 | 1534 | 2146 | 1201 | 1855 | 2298 | 1445 | 1036 | 1377 | 1698 | 2004 |

1-10 = selected spectral bands
