# Supplementary material for: Evaluating the suitability of hyper- and multispectral imaging to detect foliar symptoms of the grapevine trunk disease Esca in vineyards
Source: Plant Methods. 2020 Oct 21;16:142. doi: 10.1186/s13007-020-00685-3 (PMC7579826; doi:10.1186/s13007-020-00685-3)
Supplement: Supplementary file 3 — Additional file 3: Table S2. Results of the different machine learning approaches for the 1 year disease detection approaches. [file 13007_2020_685_MOESM3_ESM.docx]

Additional Table 2: Results of the different machine learning approaches for the one-year disease detection approaches.

|  |  | **Classification Accuracy** | | | | | | **True Positive Rate** | | | | | | **False Positive Rate** | | | | | |
| --- | --- | --- | --- | --- | --- | --- | --- | --- | --- | --- | --- | --- | --- | --- | --- | --- | --- | --- | --- |
|  |  | **VNIR** | | | **SWIR** | | | **VNIR** | | | **SWIR** | | | **VNIR** | | | **SWIR** | | |
|  |  | **2016** | **2017** | **2018** | **2016** | **2017** | **2018** | **2016** | **2017** | **2018** | **2016** | **2017** | **2018** | **2016** | **2017** | **2018** | **2016** | **2017** | **2018** |
| Symptomatic (original) | LDA | 69 | 67 | 71 | **73** | 71 | 74 | 71 | 73 | 66 | 70 | 71 | 71 | 34 | 40 | 25 | 34 | 28 | 22 |
|  | PLS | 70 | 68 | 71 | 62 | 72 | 75 | 75 | 73 | 66 | 62 | 72 | 70 | 37 | 38 | 24 | 38 | 29 | 21 |
|  | MLP | **73** | **70** | 73 | 62 | 77 | 74 | 71 | 73 | 71 | 63 | 82 | 73 | 29 | 32 | 24 | 40 | 27 | 24 |
|  | rRBF | 69 | 67 | **77** | 66 | **81** | **80** | 70 | 71 | 72 | 66 | 82 | 74 | 32 | 36 | 22 | 34 | 26 | 20 |
| Symptomatic (annotated) | LDA | 91 | 85 | 91 | 86 | 88 | 89 | 82 | 84 | 88 | 73 | 84 | 83 | 0 | 14 | 6 | 2 | 7 | 6 |
|  | PLS | 90 | 85 | 91 | 86 | 88 | 89 | 84 | 84 | 88 | 73 | 83 | 83 | 3 | 14 | 6 | 2 | 6 | 6 |
|  | MLP | **92** | **90** | **94** | 86 | 87 | 87 | 89 | 90 | 93 | 74 | 84 | 84 | 0 | 11 | 5 | 3 | 11 | 10 |
|  | rRBF | 90 | 88 | 92 | **88** | **95** | **92** | 84 | 89 | 91 | 86 | 90 | 100 | 4 | 12 | 7 | 2 | 6 | 5 |
| Pre-symptomatic | LDA | - | 58 | 73 | - | 63 | 80 | - | 55 | 75 | - | 59 | 83 | - | 39 | 29 | - | 34 | 23 |
|  | PLS | - | 59 | 73 | - | 63 | 81 | - | 52 | 75 | - | 59 | 84 | - | 35 | 30 | - | 33 | 23 |
|  | MLP | - | **61** | 75 | - | 64 | 78 | - | 63 | 78 | - | 68 | 78 | - | 35 | 28 | - | 40 | 23 |
|  | rRBF | - | 62 | **79** | - | **74** | **86** | - | 57 | 83 | - | 78 | 87 | - | 36 | 28 | - | 32 | 21 |

Best machine learning approaches were chosen according to their classification accuracy (highlighted in bold). LDA = Linear Discriminance Model, PLS = Partially Least Square, MLP = Multi-Layer Perceptron Network, rRBF = Radial-Basis Function Network
