# Supplementary material for: Evaluating the suitability of hyper- and multispectral imaging to detect foliar symptoms of the grapevine trunk disease Esca in vineyards
Source: Plant Methods. 2020 Oct 21;16:142. doi: 10.1186/s13007-020-00685-3 (PMC7579826; doi:10.1186/s13007-020-00685-3)
Supplement: Supplementary file 4 — Additional file 4: Table S3. Results of the different machine learning approaches for the model transferability evaluation. [file 13007_2020_685_MOESM4_ESM.docx]

Additional Table 3: Results of the different machine learning approaches for the model transferability evaluation.

|  |  | **Classification Accuracy** | | | | | | **True Positive Rate** | | | | | | **False Positive Rate** | | | | | |
| --- | --- | --- | --- | --- | --- | --- | --- | --- | --- | --- | --- | --- | --- | --- | --- | --- | --- | --- | --- |
|  |  | **VNIR** | | | **SWIR** | | | **VNIR** | | | **SWIR** | | | **VNIR** | | | **SWIR** | | |
|  |  | **16/17** | **16/18** | **17/18** | **16/17** | **16/18** | **17/18** | **16/17** | **16/18** | **17/18** | **16/17** | **16/18** | **17/18** | **16/17** | **16/18** | **17/18** | **16/17** | **16/18** | **17/18** |
| Symptomatic (original) | LDA | 64 | 66 | 68 | 62 | 64 | 70 | 67 | 68 | 65 | 62 | 62 | 67 | 40 | 36 | 30 | 38 | 34 | 27 |
|  | PLS | 65 | 67 | 68 | 62 | 64 | 71 | 70 | 70 | 70 | 65 | 59 | 66 | 39 | 36 | 34 | 41 | 32 | 25 |
|  | MLP | 66 | 68 | 69 | 63 | **73** | 70 | 66 | 68 | 70 | 63 | 67 | 71 | 33 | 32 | 33 | 38 | 25 | 30 |
|  | rRBF | **68** | **72** | **72** | **72** | 66 | **79** | 71 | 73 | 72 | 79 | 65 | 79 | 29 | 32 | 22 | 36 | 33 | 25 |
| Symptomatic (annotated) | LDA | 78 | 83 | 86 | 77 | 77 | 85 | 82 | 84 | 88 | 79 | 77 | 79 | 0 | 14 | 6 | 2 | 7 | 6 |
|  | PLS | 79 | 84 | 86 | 77 | 77 | 85 | 81 | 84 | 85 | 79 | 79 | 78 | 23 | 16 | 12 | 24 | 24 | 8 |
|  | MLP | **86** | **89** | **89** | 77 | **86** | 84 | 86 | 89 | 90 | 75 | 82 | 81 | 18 | 12 | 10 | 21 | 13 | 12 |
|  | rRBF | 82 | 85 | 88 | **86** | 76 | **93** | 82 | 84 | 89 | 83 | 77 | 90 | 19 | 13 | 12 | 14 | 24 | 6 |

Best machine learning approaches were chosen according to their classification accuracy (highlighted in bold). LDA = Linear Discriminance Model, PLS = Partially Least Square, MLP = Multi-Layer Perceptron Network, rRBF = Radial-Basis Function Network
